# Predicting Antigenicity of Influenza A Viruses Using biophysical ideas

**DOI:** 10.1038/s41598-019-46740-5

**Published:** 2019-07-15

**Authors:** Abdoelnaser M. Degoot, Emmanuel S. Adabor, Faraimunashe Chirove, Wilfred Ndifon

**Affiliations:** 1 Research Department, African Institute for Mathematical Sciences, Next Einstein Initiative, Kigali, Rwanda; 2 University of KwaZulu-Natal, School of Mathematics, Statistics and Computer Science, Pietermaritzburg, 3209 South Africa; 3 Research Centre, African Institute for Mathematical Sciences, Cape Town, 7945 South Africa; 4 DST-NRF Centre of Excellence in Mathematical and Statistical Sciences (CoE-MaSS), Gauteng, Wits 2050 South Africa

**Keywords:** Computational models, Applied mathematics

## Abstract

Antigenic variations of influenza A viruses are induced by genomic mutation in their trans-membrane protein HA1, eliciting viral escape from neutralization by antibodies generated in prior infections or vaccinations. Prediction of antigenic relationships among influenza viruses is useful for designing (or updating the existing) influenza vaccines, provides important insights into the evolutionary mechanisms underpinning viral antigenic variations, and helps to understand viral epidemiology. In this study, we present a simple and physically interpretable model that can predict antigenic relationships among influenza A viruses, based on biophysical ideas, using both genomic amino acid sequences and experimental antigenic data. We demonstrate the applicability of the model using a benchmark dataset of four subtypes of influenza A (H1N1, H3N2, H5N1, and H9N2) viruses and report on its performance profiles. Additionally, analysis of the model’s parameters confirms several observations that are consistent with the findings of other previous studies, for which we provide plausible explanations.

## Introduction

Currently, statistics on morbidity and mortality associated with the circulating influenza viruses are alarming and indicate the viruses represent a threat to public health. Annually around the globe, influenza viruses cause about 3 to 5 million cases of morbidity, and 250 to 500 thousand deaths^1^, with almost incalculable socio-economic costs. Among the three types of the circulating influenza viruses, namely A, B, and C, the former two types are the most prevalent and cause epidemic outbreaks nearly every year, whereas the latter type is less predominant and only causes a mild infection^2^.

Type A influenza viruses are divided into subtypes according to the combination of their two surface glycoproteins: Hemagglutinin (HA) and Neuraminidase (NA) proteins. At present, 18 and 11 variants of HA and NA, respectively, are found in animal species^3,4^. A convenient nomenclature for influenza A subtypes is based on the combination of these two proteins found on a given virus. Examples include H1N1, H3N2, H5N1 and H9N2.

Influenza viruses evolve continuously through rapid amino acid mutations of HA protein, which enables them to circumvent neutralization by antibodies that are generated by the immune system of the host as results of infections or vaccinations^4,5^. This mutational process is known as *antigenic drift*^2^, and produces new variants that are immunologically different from the parent viruses. Thus, antibodies or vaccines developed during previous infections or vaccinations do not fully recognize the new variants. Another less frequent but more malignant mutational process has also been found to be causative of antigenic variation; namely the re-assortment process, a phenomenon called *antigenic shift*, in which two or more distinct influenza viruses, possibly from different species, interchange their genetic material and generate a new variant that is completely novel to the pre-existing immunological surveillances, and that can lead to pandemic situations^2,6^.

The genome of influenza A virus comprises 8 genetic segments^4^, two of which encode for the important trans-membrane envelope proteins HA and NA. The HA protein itself is divided into two domains: HA1 (of 329 amino acids long for H3N2) and HA2 (175 amino acids for H3N2)^7^. These two domains are interconnected via covalent disulfide bounds^8^. HA1 contains five antibody-binding regions or epitopes (labelled A, B, C, D, and E) on its globular head that is used to inject the virus into the host cells through the sialic acid receptor binding sites^6^. These epitopes have been found to be the primary targets of neutralizing antibodies and regularly mutate^5^. The NA protein also undergoes some mutations but this has limited impact on virus antigenicity^9^.

Prediction of antigenic relationships (similar or variant) among influenza viruses is useful in designing (or updating the existing) effective influenza vaccines, provides important insights into the evolutionary mechanisms that underpin their antigenic variations due to the pressure of natural selection imposed by the neutralizing antibodies^10^, and helps to understand the epidemiology of these pathogens.

Two biochemical assays are commonly used to characterize antigenic relationships among influenza viruses; namely, the Hemagglutinin-Inhibition (HI) assay, which measures the ability of an antibody that has been raised against one variant to inhibit agglutination of red blood cells by another variant, and the virus micro-neutralization (MN) assay^2,11^. These serological assays are confronted with a multitude of issues including that they are labour-intensive and time-consuming; they produce variable outputs^12,13^ especially when the experiment is carried out under different conditions, such as using different concentrations of virus and of red cell^14^; they are unsuitable for large quantitative assays^15^; their outputs are contaminated by noise^14^; and they require a high level of biosafety when analyzing some viruses (e.g. H5)^16^.

A large amount of genomic data related to influenza viruses has been generated from projects like Influenza Virus Resource^17^, obtained from the high-throughput assays of complete genome sequencing made possible by new biotechnologies. Therefore, computational methods have been introduced to analyse the viral mutations. Several sophisticated computer models, arising from different theoretical perspectives, have been proposed which combine high-throughput data and low-throughput antigenic data (e.g. from HI assays) to study virus antigenicity. Some models infer antigenic relationships among influenza variants (predictive models) from the amino acid mutations from large numbers of sequences on HA1 domains, while others make predictions about the flu variants that will dominate the next season (forecasting models) from the surveillance data of the current season^18^. However, the prediction step precedes the forecasting step because the vaccine only has to be updated if the newly emerging variant differs antigenically from the current vaccine strain. Also, from the results of a prediction model, antigenic cartography- grouping influenza viruses into distinct clusters- and phylogenetic tree of influenza viruses could be established, which facilitate visualization and interpretation of antigenic relationships. Through construction of a 2D antigenic map, using a multidimensional scaling technique, Smith *et al*.^15^ characterized the antigenic evolution history for influenza A/H3N2 viruses from 1968 to 2003. Likewise but using a 3D instead of a 2D antigenic map, Barnett *et al*.^19^ developed an online antigenic cartography resource to determine antigenic drifts and shifts for influenza viruses. Liu *et al*.^20^ combined phylogenetic trees with a Naïve Bayesian Model to map the antigenic patterns of influenza A/H1N1 viruses in China. Based on phylodynamics analysis Hadfield *et al*. developed an online application called *Nextstrain* for real-time tracking and visualization of the evolution of influenza and other epidemiological viruses such as Zika and Ebola^21^.

The prediction models that correlate genetic mutation and antigenic data were developed based on various inference techniques, including information theory^12^ and data-driven machine-learning approaches^13^. Liao and his co-workers^7^ proposed a number of bioinformatics models, which are based on scoring models and regression methods including multiple regression, logistic regression and Support Vector Machine (SVM), for predicting antigenic variants of influenza A/H3N2 viruses. Moreover, Lees *et al*.^10^ developed several linear models accounting for different regions on HA1 sequence including the five epitopes, N-linked glycosylation, and other non antigenic regions, for predicting the antigenicity of A/H3N2 strains. Yin *et al*.^22^ developed a staking model that draws a consensus conclusion from the outcomes of set of classifiers. Furthermore, some prediction methods do not rely solely on the number of amino acid changes between two variant sequences, but also incorporate additional properties such as amino acids substitution metrics^13^ that account for certain features of amino acid residues like BLOSUM62, or physiochemical differences like the amino acid volume and electrostatic charge^8,23^, in order to improve the predictive performance. Zhou *et al*.^24^ proposed a Context-Free Encoding Scheme (CFreeEnS) prediction method that allows to be integrated with a large number of different substitution matrices for protein sequences.

These computational methods have already demonstrated their usefulness, helped to uncover many characteristics of virus evolution, and provided a promising approach for efficient vaccine strains selection^2^. However, a majority of these prediction models are data driven and lack a concrete and mechanistic theoretical basis.

In this study we present a simple biophysical model that can predict antigenic relationships among influenza A viruses using both genomic amino acid sequences and experimental antigenic data. Antigenicity of an influenza virus has two remarkable features: sparsity- a few critical positions on the HA1 protein undergo antigenically consequential genetic mutation, and co-evolution- certain positions tend to co-mutate jointly^25^. Therefore, we enhance our model by carefully choosing a regularization term that actualizes both features, namely the elastic-net lasso. We demonstrate the applicability of the model using benchmark datasets of four subtypes of influenza A viruses (H1N1, H3N2, H5N1, and H9N2) and report on its performance profiles. Furthermore, we checked the effectiveness of model on a large, novel (unseen) dataset of influenza A/H1N1 viruses and found that model achieved a high AUC value (an established measure of prediction performance) of 0.86.

## Materials and Method

### Materials

The model has been developed using both antigenic data and HA1 protein sequences of influenza viruses. The antigenic data that measures the relationship between pairs of influenza viruses are given in the form of reciprocal normalized Hemagglutinin Inhibition-(HI) values, denoted NHT^14^. The smaller the NHT value the closer the antigenic similarity between the two viruses. A pair of viruses is considered to be antigenically similar if their corresponding log-transformed NHT value is ≤log(4), and otherwise they are said to be antigenically different^25^. We obtained a total of 1557 pairs of influenza A viruses with measured antigenic relationships, spanning 4 subtypes, from the study of Peng *et al*.^12^, which is publicly available and has been assembled from the relevant literature and documents published by the collaborating networks of the World Health Organization’s (WHO) global influenza surveillance network^26^ (for more information, see the Supplementary Material of^12^). These datasets have been partially used in the study of other computational methods in this research area^8,10^. We choose these particular datasets because of their epidemiological importance and also we would be able to readily make comparisons with other models that have used them. The datasets contained information for 291 unique influenza A viruses.

We downloaded the HA1 protein sequences of the 291 viruses belonging to the 4 subtypes from the Influenza Virus Resource^17^. For each subset of HA1 proteins belonging to a particular subtype, we performed multiple sequence alignment analysis using the msa package in R^27^. Additionally, 131 important amino acid positions found on or nearby the five canonical epitopes of the H3N2 viruses, which are the primary targets of the neutralizing antibodies, were obtained from the literature^5,8,10,12^. These positions are given in Table S1 in the Supplementary Materials. Table 1 below provides an overview of the datasets used to build the model.

**Table 1:** Overview of antigenic datasets used to develop the model.

Subtypes	^#^Viruses	^#^Pair viruses	^#^Similar viruses	% of Similar viruses
H1N1	67	355	163	46%
H3N2	139	791	382	48%
H5N1	56	293	113	39%
H9N2	29	118	31	26%
Total	291	1557	689	Average: 40%

### Model

The mathematics of statistical mechanics defines the relationship between the energy of a system such as the binding of a virus to an antiserum, and its thermodynamic quantities, which in our case are represented by NHT measurements. If the functional forms specifying the energy (Hamiltonians) are known, we can derive the thermodynamic measurements, and vice-versa. In complex systems, like the virus-antiserum mixtures we deal with here, it is difficult to identify the Hamiltonians precisely, but they could be approximated from thermodynamic measurements. Here we take the inverse approach: from the available empirical measurements we approximate the Hamiltonians that govern the interactions between the virus and antiserum, through a machine learning approach. In fact, recently, several problems in computational biology have been addressed by means of such an inverse biophysical approach^28–31^, with very meaningful results.

This inverse approach cannot be applied directly in the case of antigenic similarity among influenza viruses because three elements are involved in the process of determining the HI value: the two viruses, [the homologous virus (*ν*_*i*_) and the heterologous virus (*ν*_*j*_)], and the antiserum (*Y*). However, analogous to the steps of serological assays, the quantification of the HI value can be viewed as follows: A concentrated solution of the homologous virus *ν*_*i*_ and red cells is mixed up with the antiserum *Y* under standard conditions. This mixture of virus-antiserum attains an equilibrium state^14^. Suppose that its energy is given by *E*(*ν*_*i*_). Likewise, under the same conditions, suppose a concentrated solution of the heterologous virus *ν*_*j*_ is mixed up with the same red cells and antiserum, and reaches the equilibrium state with energy *E*(*ν*_*j*_). Then one could define the antigenic relation between the two viruses as a function of the difference between the two energies, Δ*E*_*ij*_ = *E*(*ν*_*i*_) − *E*(*ν*_*j*_). Thus, we compute the antigenic dissimilarity or “distance” between two strains, say *ν*_*i*_ and *ν*_*j*_, of influenza viruses as follows:

1 $${d}_{ij}={e}^{{\rm{\Delta }}{E}_{ij}}\Rightarrow {\rm{\Delta }}{E}_{ij}=\,\mathrm{log}({d}_{ij})$$

Now, assuming that energy is additive, let us assign a Hamiltonian function for each amino acid type at each position of the sequence of HA1 protein for the particular virus (refer to the Supplementary Materials for full details about the formulation of the model) such that Δ*E*_*ij*_ is the sum of changes in energy between the two strains and it is computed as follows:

2 $${\rm{\Delta }}{E}_{ij}=\sum _{s=1}^{n}\,\delta H({a}_{s(k)}^{i},{b}_{s(\bar{k})}^{j}),$$

where $\delta H({a}_{s(k)}^{i},{b}_{s(\bar{k})}^{j})$ is the function or Hamiltonian that assigns an amount of energy required when amino acid *a* of type *k* on virus *ν*_*i*_ changed to amino acid *b* of type $\bar{k}$ on virus *ν*_*j*_, both at position *s*. *k* and $\bar{k}$ could be any of the 20 standard amino acids, and *n* is the length of the HA1 protein. This definition of antigenic similarity can potentially tolerate sub-optimal matching between pairs of sequences being compared.

Notice that the definition of the antigenic relatedness in Eq. (1) is consistent and well-defined; because *d*_*ij*_ = 0 for perfectly similar viruses i.e. *ν*_*i*_ = *ν*_*j*_, and the *d*_*ij*_ increases as the two viruses become more dissimilar/divergent. The Hamiltonians vector *δH* represents the model’s parameters and is learned from the experimental measurements (NHT), by minimizing the following mean square error (MSE):

3 $${MSE}=\frac{1}{N}\sum _{n=1}^{N}\,{[{D}_{ij}^{(n)}-{d}_{ij}^{(n)}]}^{2}$$

where *D*_*ij*_ is the target value between viruses *i* and *j*, derived from the log-transformed NHT measurements, log(*HI*_*ij*_). Equation (3) is subject to the following constraints:

4 $${P}_{\alpha }(t)=\sum _{j=1}^{d}\,[(1-\alpha )\delta {H}_{j}^{2}+\alpha |\delta {H}_{j}|]\le t$$

for regularization parameters *t* > 0 and 0 ≤ *α* ≤ 1. This constraint in Eq. (4) represents the penalty term of the model; it is a combination of *L*_1_ and *L*_2_, which is known as elastic-net lasso^32^. *L*_2_ is good at detecting correlations among the features. In this context we devise it to capture the co-evolving positions. Meanwhile *L*_1_ induces sparsity into the model, consistent with empirical expectations. Also the regularization term P_*α*_(t) avoids data over-fitting and improves the model’s performance on a novel dataset. *t* is the meta-parameter that governs the whole penalization term. *α* is a trade-off between *L*_1_ and *L*_2_; the larger the value of *α* the greater the emphasis on *L*_1_, and the smaller the value of *α* the greater the emphasis on *L*_2_.

Equation (3) together with the penalization term of Eq. (4) form a non-linear but convex function, and we solved it via an iterative, cyclic coordinate descent and adaptive Gauss-Seidel method. Details of this optimization algorithm are found in^33^.

## Results

### Predictive performance of the model

We evaluated the predictive performance of the model on datasets for the four influenza A subtypes considered in this study (Section 1.1), using a five-fold cross-validation test to avoid data over-fitting. In the cross-validation process, an antigenic dataset of a particular subtype was randomly divided into five quasi equal-sized subsets. We held-out one subset, while the remaining four subsets were combined and used to train the model. The held-out subset was used to test model performance. This process was repeated for each subset, and the test results from all the five subsets were combined in order to assess the overall performance of the model subject to that particular subtype.

We used several statistical metrics to measure the model’s performance, including Area Under the Curve (AUC) of the Receiver-Operator Characteristic (ROC), accuracy, sensitivity, specificity, Pearson correlation, and root mean square error (RMSE). The AUC is a measure that takes both true positive rate (TPR) and false positive rate (FPR); its value ranges from 0 to 1. The higher the AUC value, the better the performance, and the AUC value of = 0.5 is equivalent to the performance of a random model. The accuracy metric assesses the ability of the model to correctly identify the antigenic similarity between two influenza viruses.

**Figure 1:**
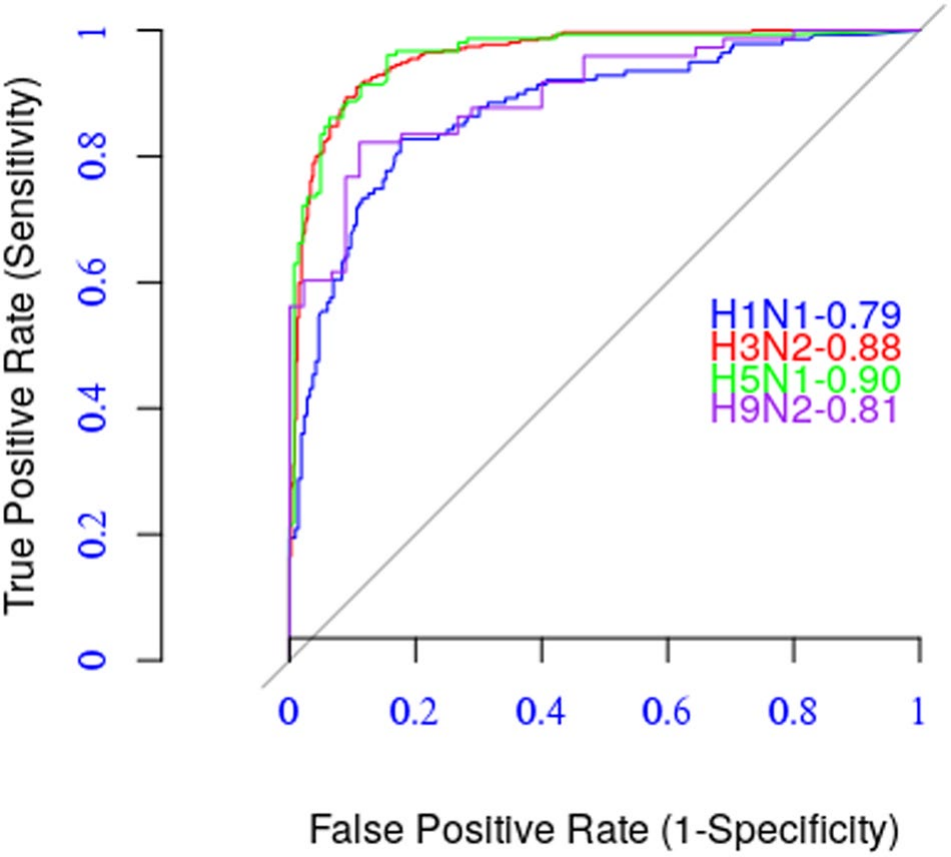
ROC Curves.

Table 2 shows the relative performance of the model based on these metrics, and Fig. 1 depicts ROC curves for the four influenza A subtypes.

**Table 2:** Five-fold cross-validation results of the model.

Metrics	AUC	Accu	Sens	Spec	Cor	RMSE
H1N1	0.79	0.81	0.67	0.90	0.73	0.04
H3N2	0.88	0.90	0.82	0.95	0.88	0.02
H5N1	0.90	0.89	0.86	0.93	0.86	0.05
H9N2	0.81	0.79	0.71	0.91	0.76	0.09

**Figure 2:**
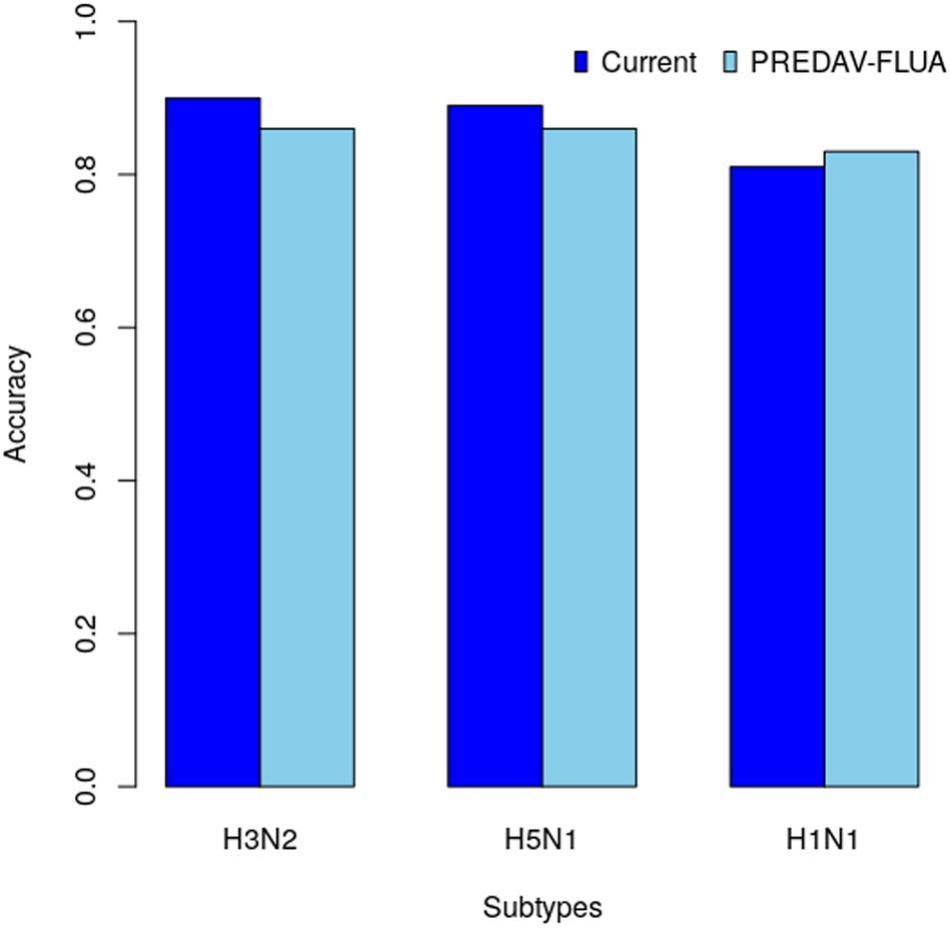
Comparing with the PREDAV-FLUA model.

The model has an excellent predictive performance for subtype H5N1 (AUC = 0.90, accuracy = 0.89, and correlation = 0.86) and a very good performance for the other three subtypes. We compared the results of the current model to the results of the residue-based model PREDAV-FluA^12^, which was built using the same datasets. We observed that the current model outperformed the PREDAV-FluA model for both H3N2 (accuracy = 0.90 to 0.86) and H5N1 (accuracy 0.89 to 0.86) subtypes. The latter model had a better performance for the H1N1 subtype (accuracy 0.81 to 0.83) (see Fig. 2).

### Energy variation across the canonical epitopes of H3N2

**Figure 3:**
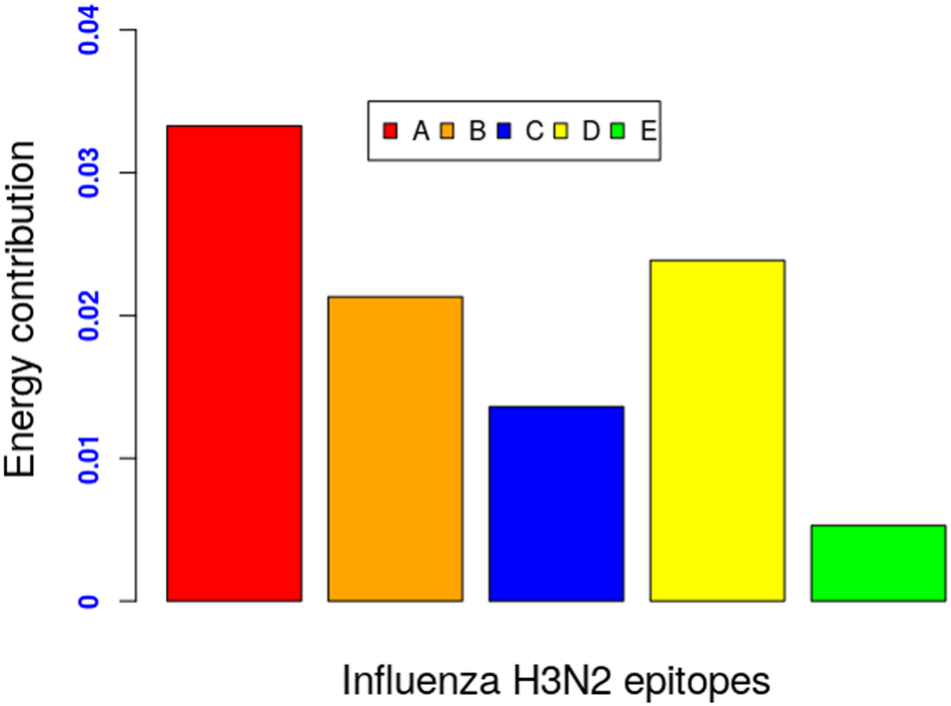
Energy distribution over epitopes.

Analysis of the model’s parameters also revealed that all five immunodominant epitopes contributed varying amounts of energy to the antigenic properties of each of the four considered influenza A subtypes. This is consistent with the long and well-established observation that correlates the antigenic variation to concurrent mutations in multiple distinct regions of the HA1 sequence^10^. Strikingly, for Influenza A H3N2 subtype, the high efficiency neutralization epitopes, A, B, and D^5^, which are located within a close spatial proximity of receptor-binding sites (see Table S3 in the Supplementary Materials) and are the most preferred targets of antibodies, contribute relatively higher amounts of energy compared to low efficiency neutralization epitopes, i.e. epitopes E and C (Fig. 3). This finding is in agreement with other previous studies^5,13,25^.

**Figure 4:**
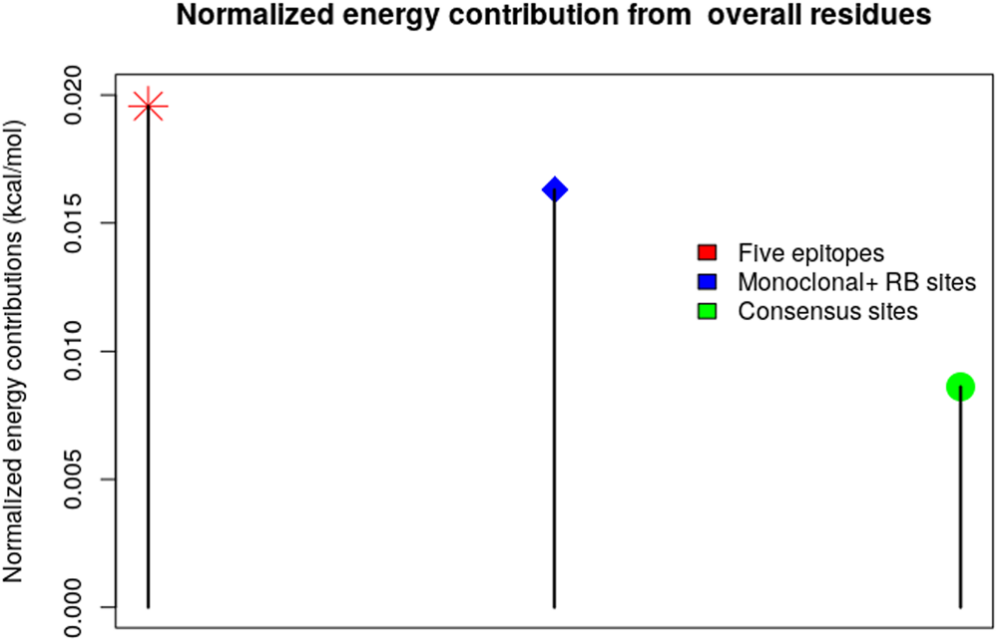
Normalized energy contribution from overall residues.

Moreover, we found that most of the energy contributions, approximately 67% of the total energy, actually arise from the five epitopes. The rest of it distributed between residues that has been found to be targets of monoclonal antibodies^5^, receptor binding (RB) sites, and other consensus sites (Fig. 4).

### Influenza A subtypes undergo similar mutations

**Figure 5:**
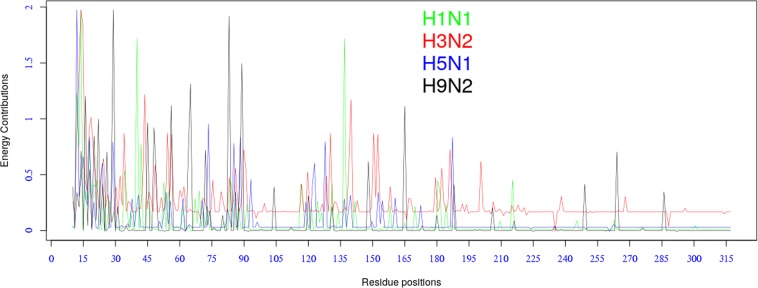
Energy distribution over residue positions.

It is generally considered that genomic variation in the HA1 protein drives the evolution of influenza viruses and allows them to escape antibody neutralization. From the analysis of the model’s parameters we found that the four influenza A subtypes (H1N1, H3N2, H5N1, H9N2) exhibit similar patterns of energy fluctuations over HA1 protein positions. As shown in Fig. 5, the four subtypes share very similar energy peaks over certain positions along the HA1 sequence. This observation indicates the existence of a common evolution mechanism among influenza A viruses, and supports the conclusions of other previous studies^13,34,35^. The energy contribution of each position was calculated by summing up the residue-residue Hamiltonians associated with that position (See Eq. 1 in the Supplementary Materials).

Notice that the high peaks in Fig. 5 reflect strong repulsive energies, i.e mutations that can cause antigenic drift events (positively selected positions), while the low peaks reflect attractive energies, i.e. mutations that cause viruses to become antigenically more similar.

Most positions (64%, 76%, 64% and 71% of H1N1, H3N2, H5N1 and H9N2 positions, respectively) are silent, i.e. with zero energy contributions, consistent with the empirical fact that only minority of positions undergo antigenically consequential mutations.

In addition, we also found strong correlations among influenza A subtype Hamiltonians; varying from 0.87 between H1N1 and H3N2, with p-value = 0.026); to 0.67 between H1N1 and H95N2, with p-value = 0.01 (see Table S2 in the Supplementary Materials).

### Predictions on a validation dataset

To examine the effectiveness of the model presented here and to assess how well it can be extrapolated on a novel (unseen) dataset, we tested the predictive performance of the model on a dataset of epidemics and pandemics of influenza A/H1N1 which we obtained from ref.^22^. Similar to the dataset described in Section 1.1, the validation dataset also has two parts, antigenic data based on hemagglutination inhibition (HI) assay and genomic sequences. The HI antigenic data were compiled from various sources of flu data, including the Francis Crick Institute (FCI), European Centre for Disease Prevention and Control (ECDC), World Health Organization (WHO), U.S Food and Drug Administration (FDA), and others. The genomic sequences were obtained from Influenza Virus Resource (IVR)^36^ and Global Initiative on Sharing All Influenza Data (GISAID)^37^. The original dataset was large, but after excluding the A/H1N1 viral pairs that occurred in the dataset used to build the model and removing duplications, we ended up with a total of 642 viral pairs and 168 sequences (Table 3). We choose this dataset for a number of important reasons. Firstly, it is publicly available, organized in a chronological order, and contains antigenic data related to the last five epidemics and pandemics caused by A/H1N1 viruses, from 1921 to 2016. Secondly, other computational methods have greatly neglected influenza A/H1N1, to such a degree that there is insufficient knowledge about it^22^. Table 3 gives per period summary of the dataset, the cleaned antigenic data and genomic sequences are provided in Supplementary Files S1 and S2, respectively.

**Table 3:** The validation dataset.

Period	Year	Event	Type	# of sequences	Viral pairs
II	1921–1976	Seasonal Flu 1	EPD	31	65
III	1977–1980	Russian Flu1 977	PDM	35	139
IV	1981–2008	Seasonal Flu 2	EPD	59	332
V	2009–21011	Swine 2009	PDM	30	90
VI	2012–2106	Seasonal Flu 3	EPD	13	16

**Figure 6:**
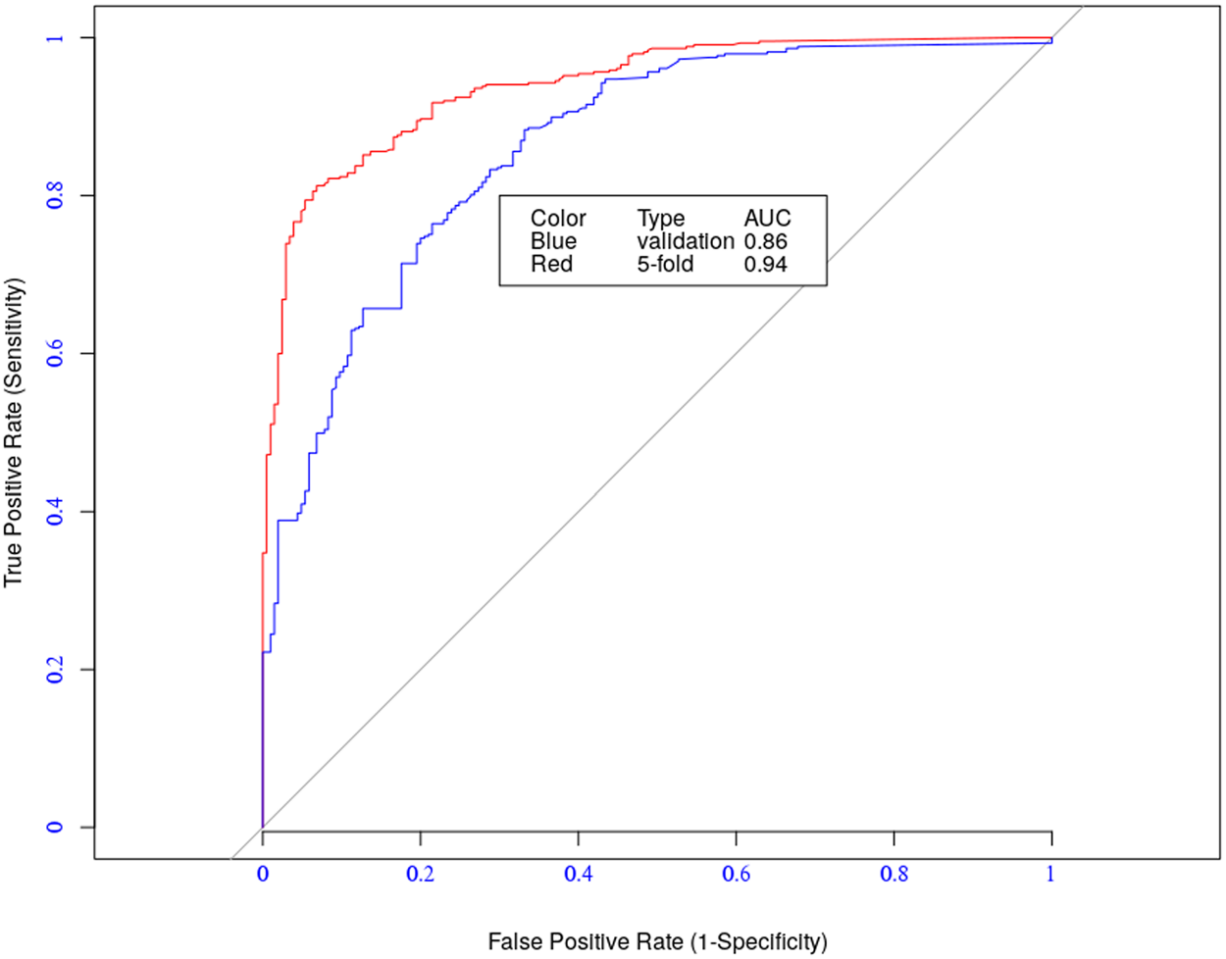
Energy distribution over residue positions.

The predictive performance of the model on the validation dataset was assessed using two different strategies. In the first strategy, we utilized the validation dataset as a novel dataset and measured the performance using the model’s parameters learned using from the A/H1N1 data described in Section 1.1. The model achieved an AUC value of 0.86 (blue curve in Fig. 6). In the second strategy, we performed a five-fold cross-validation test using the validation dataset itself. In this case the model achieved an AUC value of 0.94 (red curve in Fig. 6). In the later case, the model has an improved performance because of the larger size of the dataset. These results demonstrate the reliability of the model in predicting antigenic relationships.

### General model

The model given by Eq. (3) together with the penalization term of Eq. (4) is a subtype model; thus it was applied for each individual influenza subtype. But, we thought of a general model that predicts the antigenicity of all influenza subtypes, based on the simple fact that influenza viruses share a common evolutionary origin. In this section, we show that a model can be upgraded to become a general model.

From the available crystallographic structures of influenza A viruses, ten antigenic regions on the HA1 proteins; called artificial sites and largely govern the antigenic variations, has been identified^10,12^. These regions; denoted by *E*_1_ to *E*_10_, cover almost the entire HA1 protein and each one contains group of amino acid residues according to the distance from the globular head of HA in a descending order. We utilized such antigenic regions to generalize our model so that it could predict antigenic similarities for all subtypes of influenza A. Thus, we changed the definition of Δ*E*_*ij*_ in Eq. (2) between two strains *ν*_*i*_ and *ν*_*j*_ as follows:

5 $${\rm{\Delta }}{E}_{ij}=\sum _{k=1}^{10}\,\delta H(k,a,b),$$

such that *δH*(*k*, *a*, *b*) accounts for the energy of replacing amino acid *a* in strain *i* by amino acid *b* in strain *j* at the antigenic region *k*. The rest of equations remain unchanged.

**Figure 7:**
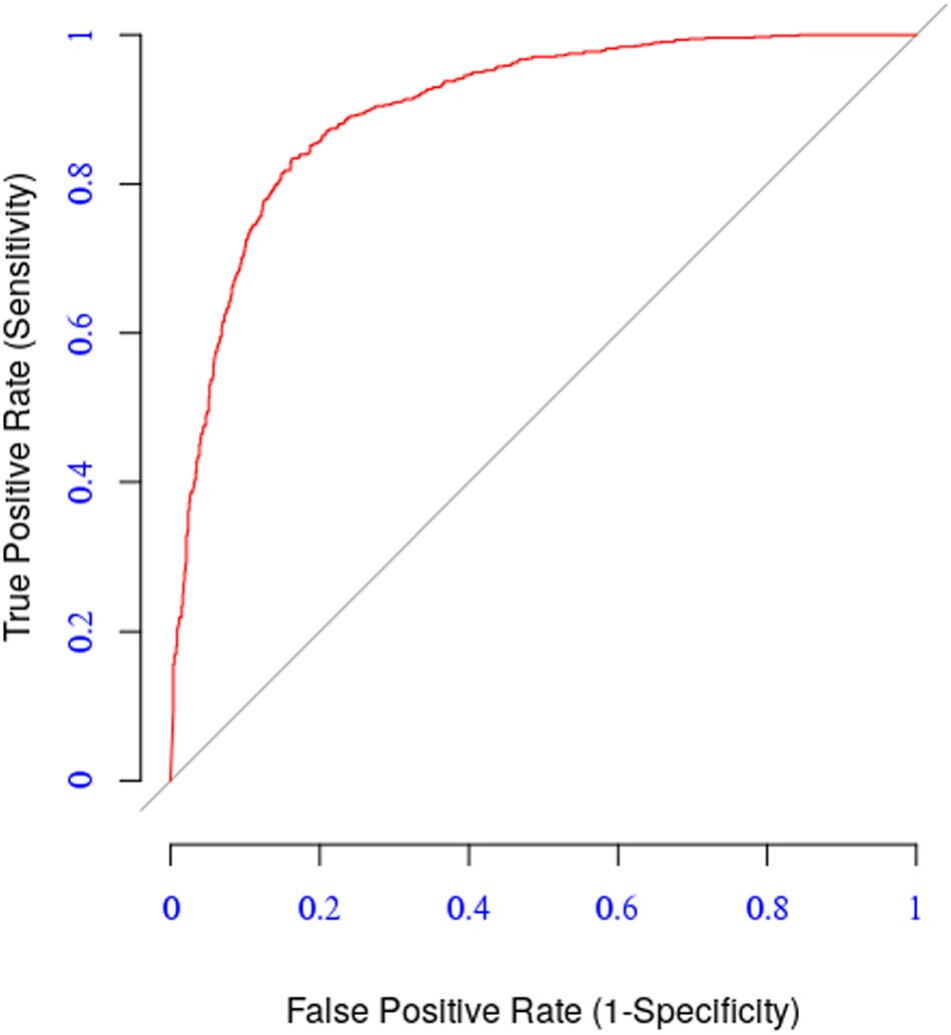
Performance of general model.

We evaluated the performance of the general model on the dataset described in Section 1.1 of all the four influenza A subtypes considered in this study, using five-fold cross-validation. It scored AUC value = 0.78 (Fig. 7), accuracy = 0.80, sensitivity = 0.65, specificity = 0.92, and correlation = 0.77.

## Discussion

Accurate and reliable prediction of antigenic similarity among influenza viruses is necessary for optimal vaccine strain selection. In this work we have presented a new approach for predicting antigenic relatedness of influenza variants based on biophysical ideas.

Many factors related to the HA1 protein influence the antigenicity of influenza viruses, spanning from structural conformations^23^ to physiochemical features^8^ like hydrophobicity; however, these factors are not independent as has been demonstrated by the work of *Yuhua et al*.^13^. The predictive performance of their model improved when they integrated the model with combination of a few substitution metrics that reflected some of those factors, especially structure-based substitution matrices. But from the properties of thermodynamic and statistical mechanics, energy is a *universal currency*^38^. Thus, it is expected that the energy would reflect the aggregated net-effect of all the factors associated with the HA1 protein that might influence the antigenicity. This extremely useful inclusion of all factors, in a very simple way, is the power of our approach over other sequence-based methods which try to incorporate a few selective factors through some amino acids substitution metrics.

Sparsity is a hallmark property of cellular processes; for example, in this context, a few amino acids mutations drive most of the antigenic drift events for influenza viruses. Therefore, our model accounts for the sparsity, with the aid of an elastic-net penalization term.

From their cartography model that characterizes the evolutionary dynamics of H3N2 viruses, Smith *et al*.^15^ identified a list of amino acid substitutions that are associated with antigenic drift events between 10 clusters of H3N2- called cluster-difference substitutions- using influenza surveillance data collected between 1968 and 2003. We further investigated the consistency of our model’s parameters with that of^15^. As expected, we found that most of these amino acid substitutions corresponded to Hamiltonians of repulsive energy. Of the 67 cluster-difference amino acid mutations, 46 (approximately About 70%) were found to have non-zero energetic contributions (Table 4). Furthermore, most of these cluster transitions are the result of several concurrent cluster-difference substitutions; for example, transition from England 1972 (EN72) to Victoria 1975 (VI75) characterized by 12 amino acid substitutions (Table 4). In such cases we found some of the substitutions contribute a positive amount (repulsive) of energy, while some of them contribute a negative amount (attractive) of energy. A few cluster transitions are the result of single amino acid mutation; for example, the two transitions from Sichuan 1987 (SI87) to Beijing 1989 (BE89) and Beijing 1992 (BE92) to Wuhan 1995 (WU95), are both characterized by only single mutation N145K (at position 145 of HA1 protein, the amino acid asparagine (N) changed to amino acid lysine (K)). In these cases, we found that such substitutions always have strong repulsive energy (see Table 4). Overall, the net energy contribution for any cluster transition is always positive.

**Table 4:** Relative energy contributions for important amino acid mutations that drive clusters transition.

Cluster transition	Amino acid substitutions and energy											
	A		B		C		D		E		O	
HK68-EN72	T122N G144D	0.024 0.00	T155Y N188D	0.136 0.007	—	—	R207K	0.005	—	—	—	—
EN72- VI75	N137S S145N	0.266 0.00	L147Q Q189K S193D	0.808 −0.028 0.00	N53D I278S	0.453 0.00	F274S R102K I213V I217V I230V	0.00 0.00 0.013 0.00 −0.021	—	—	—	—
VI75- TX77	S137Y	0.069	G158E Q164L D193N	−0.033 0.00 0.00	K50R D53N	1.451 0.00	S174F K201R V213I V230I	0.002 0.047 0.019 −0.087	E82K M260I	0.661 −0.012	—	—
TX77- BA79	N133S P143S G146S	0.331 0.04 −0.006	K155E T160K Q197 R	0.138 −0.044 −0.025	N53D N54S	0.453 0.031	D172G V217I V244L	−0.861 0.000 −0.635	K82E	0.00	—	—
BA79- SI87	G124D	0.615	Y155K K189R	0.000 0.218	—	—	—	—	—	—		
SI87- BE89	N145K	0.557	—	—	—	—	—	—	—	—	—	—
BE89- BE92	S133D K145N	0.642 1.012	E156K E190D	0.249 0.001	—	—	—	—	T262N	−0.033	—	—
BE92-WU95	N145K	0.557	—	—	—	—	—	—	—	—	—	—
WU95-SY97	—	—	K156Q E158K V196A	0.810 0.174 0.00	N276K	0.00	—	—	k62N	0.00	L25I V202I W222R G225D	1.669 −0.053 −0.060 −0.196
SY97-FU02	A131T	0.247	H155T Q156A	0.00 0.00	R50G	0.00	—	—	H75Q E83K	0.015 0.071	—	—

The model can be readily incorporated into current surveillance systems for influenza  -e.g. by supporting surveillance labs to interpret the antigenic and sequence data they routinely collect. The logical next step of this work is to extrapolate the structural similarities among influenza A viruses and develop a more accurate general purpose and pan-specific computational model that could predict the antigenicity of all the subtypes. Such a model would reconcile both structural inconsistencies among influenza viruses and experimental artefacts associated with antigenic data in order to obtain more accurate results. A further study is needed to produce a forecasting version of the model based on the same biophysical principles which would be helpful in recommending influenza vaccine strains.

## Supplementary information

Supplementary File

Dataset 1

Dataset 2
