# Supplementary material for: Predicting Antigenicity of Influenza A Viruses Using biophysical ideas
Source: Sci Rep. 2019 Jul 15;9:10218. doi: 10.1038/s41598-019-46740-5 (PMC6629677; doi:10.1038/s41598-019-46740-5)
Supplement: Supplementary file 1 — Supplementary File [file 41598_2019_46740_MOESM1_ESM.pdf]

Predicting Antigenicity of Influenza A Viruses Using biophysical ideas
Supplementary Information

A. M. Degoot, Emmanuel S. Adabor, Faraimunashe Chirove and Wilfred Ndifon

June 4, 2019

Hemagglutinin Inhibition

Hemagglutinin Inhibition (HI) assay is the preferred biological assay for determining antigenic similarity among influenza viruses, and it is recommended by World Health Organization (WHO) in vaccine formulation [1]. Antigenic similarity of virus v_1 relative to virus v_2 is defined as the ability of antibodies designed against virus v_2 to neutralize virus v_1, and its not always symmetric [2].

The antigenic distance between a pair of viruses was computed as follows [1, 2]:

$$D_{ij} = \log\left(\sqrt{\frac{H_{ii} \times H_{jj}}{H_{ij} \times H_{ji}}}\right)$$

where H_{ij} is the maximum dilution of antiserum containing antibodies raised against virus j that can block virus i from agglutinating red cells.

Model formulation

Given two influenza viruses, say v_1 and v_2, of the same subtype with measured antigenic similarity $d_{1,2}$ given as defined in Section . Let us consider the amino acid sequences of the hemagglutinin (HA1) of the two viruses as follows:

$$v_1 = A_1 A_2 A_3 \ldots\ldots\ldots A_i \ldots\ldots A_n$$
$$v_2 = B_1 B_2 B_3 \ldots\ldots\ldots B_i \ldots\ldots B_n,$$

where A_i (and similarly for B_i), $1 \leq i \leq n$, is amino acid residue at position i, and n is the length of the virus sequence, which is the the monomer of HA_1 for the considered subtypes, for example $n = 329$ for H3N2. Both viruses are assumed to have a unified length; at least after sequence alignment.

Assume that two concentrated solutions of virus and red cells for both viruses have been mixed separately with the same antiserum (vaccine) containing antibodies Y. These mixtures of virus-antiserum attain equilibrium states[2]. Consider the equilibrium states of the reactions representing the two mixtures as follows:

$$v_1 + Y \leftrightarrow v_1 Y$$
$$v_2 + Y \leftrightarrow v_2 Y.$$

These two states describe macro-states for reactants and solvents.

Suppose there is a function or Hamiltonian H_i^1 (and similarly H_i^2) that specifies energy for amino acid residue of type $A_i(B_i)$ at position i of variant $v_1(v_2)$ within the solvent $v_1Y(v_2Y)$:

$$H_i^1 : \{\text{AA residue}\} \longrightarrow R$$

and similarly

$$H_i^2 : \{\text{AA residue}\} \longrightarrow R$$

Assume the Hamiltonian are additive, then the total energy of solvent $v_1 Y$ is

$$E(v_1|Y) = \sum_{i=1}^{n} H_i^1(A_i)$$

and similarly for $v_2 Y$ is

$$E(v_2|Y) = \sum_{i=1}^{n} H_i^2(B_i).$$

Now, we calculate the difference of energy between the mixtures as follows:

$$\Delta E = E(v_1|Y) - E(v_2|Y) = \sum_{i=1}^{n} H_i^1(A_i) - \sum_{i=1}^{n} H_i^2(B_i) = \sum_{i=1}^{n} \left[H_i^1(A_i) - H_i^2(B_i)\right] = \sum_{i=1}^{n} \delta H_i(A_i, B_i)$$

such that $\delta H_i(A_i, B_i)$ is a function that assigns the difference of energy value (Hamiltonian) between residue of type A_i on v_1 sequence and residue of type B_i on v_2 sequence, both at position i.

In this way, for any given pair of viruses we can construct a vector with each entry either: 1 if it contains amino acid of type A_i on v_1 sequence and amino acid of type B_i on v_2 sequence, at position i, or 0. The dimension of the vector $= n * 20 * 20$, accounting for all possible 20 amino acids on both viruses, and the length HA1 n.

For simplicity, in this model we considered the informative sites (positions which do mutate), although the mutational and co-mutational effects of non-informative sites (invariant positions which do not evolve) might echo in other sites and change their energetic contributions. Therefore, $\delta H_i(A_i, B_i) = 0$ wherever $A_i = B_i$.

Also assume the antigenic similarity $d_{1,2}$ is some function of the two mixtures $v_1 Y$ and $v_2 Y$,

$$d_{1,2}(v_1, v_2|Y) = f : (v_1, v_2) \longrightarrow R.$$

we choose f to be an exponential function

$$d_{1,2} = e^{\Delta E}.$$

Again for simplicity and numerical stability we take the log-transformation

$$\Delta E = \log(d_{1,2}).$$

Position energy contribution

The energy contribution of each residue position; $i = 1, 2, \dots n$ along the HA1 protein is computed as follows:

$$E(i) = \sum_{a,b \in \Phi} \delta H(a^i, b^i) \tag{1}$$

where Φ is the set of the 20 standard amino acids.

Mutational energy

Pairwise mutational energy, E_μ, between two residues, say a and b, is computed as follows:

$$E_\mu(a, b) = \sum_{i=1}^{n} \delta H(a^i, b^i) \tag{2}$$

where Φ is the set of all the 20 standard amino acids.

Supplementary tables

Important HA1 sites found on the five canonical positions

**Epitope**	**# sites**	**Amino acid sites**
A	19	122, 124, 126, 130, 131, 132, 133, 135, 137, 138, 140, 142, 143, 144, 145, 146, 150, 152, 168
B	22	128, 129, 155, 156, 157, 158, 159, 160, 163, 164, 165, 186, 187, 188, 189, 190, 192, 193, 194, 196, 197, 198
C	27	44, 45, 46, 47, 48, 50, 51, 53, 54, 273, 275, 276, 278, 279, 280, 294, 297, 299, 300, 304, 305, 307, 308, 309, 310, 311, 312
D	41	96, 102, 103, 117, 121, 167, 170, 171, 172, 173, 174, 175, 176, 177, 179, 182, 201, 203, 207, 208, 209, 212, 213, 214, 215, 216, 217, 218, 219, 226, 227, 228, 229, 230, 238, 240, 242, 244, 246,247,248
E	22	96, 102, 103, 117, 121, 167, 170, 171, 172, 173, 174, 175, 176, 177, 179, 182, 201, 203, 207, 208, 209, 212, 213, 214, 215, 216, 217, 218, 219, 226, 227, 228, 229, 230, 238, 240, 242, 244, 246,247,248
O	22	49, 60, 74, 79, 90, 274, 151, 52,277, 220, 134, 136, 153, 17, 199, 2, 3,4, 31, 112, 205, 271
R	7	98, 136, 153, 183, 195, 224, 225

Table S1: Lists of important amino acid sites of HA1 found on each of the five individual epitopes (A, B, C, D and E); O (Others) is list of sites that do not belong to any of the five epitopes, but are under positive selection of monoclonal antibodies [3, 4]; and R refers to sites found on the receptor-binding region. These lists were collected from the references [3, 5, 6, 7].

Correlations and p-values

Subtypes	H1N1	H3N2	H5N1	H9N2
H1N1	-	0.87	0.85	0.67
H3N2	0.026	-	0.85	0.75
H5N1	0.001	0.0001	-	0.76
H9N2	0.01	0.0003	0.335	-

Table S2: Correlation coefficients and p-values between Hamiltonians of influenza A viruses. The upper triangle shows the correlations (Pearson) coefficients and the lower triangle shows p-values (Wilcoxon rank test).

Supplementary Table and Figure Legends

Table 1: An overview of antigenic data utilized in this study. Data were obtained for the 4 subtypes, H1N1, H3N2, H5N1, and H9N2. For each subtype, the first column shows the number of viruses, the second column gives the number of antigenic data, the third column shows the number of antigenically related pair of viruses, and the last column gives the percentage of similar viruses. The last row shows the aggregate result for each column.
Table 2: Performance of the model in five-fold cross-validation for the four influenza A subtypes. The

Energy contributions and spatial distances

Epitopes	Spatial distance (in Å)	Energy contribution (in kcal/mol)
A	10.7	0.63
B	13.0	0.47
C	44.5	0.37
D	17.7	1
E	25.5	0.12

Table S3: Energy contributions for the five canonical epitopes for H3N2 and their spatial distance for the receptor-binding site. Distance measurements were taken from the study of Ndifon et al. [3].

columns Accu, Sens, Spec, Cor, and RMSE stand for Area under curve, Accuracy, Sensitivity, Specificity, Correlation (Pearson Correlation Coefficient), and root mean square error, respectively.

Table 3: An overview of the H1N1 validation dataset for its last five epidemic (**EPD**) and pandemic (**PDM**) outbreaks.

Table 4: Energy contributions for a list of important amino acids substitutions associated with transition among 10 temporally adjacent clusters of H3N2 viruses, from 1968 to 2003 given in a chronological order. The list of the substitutions are organised according to their residue locations within the five canonical epitopes; A, B, C, D, and E; and O (others) which contains a set of immunologically important positions outside of the five epitopes. Energy contribution is given next to each substitution. The clusters and the list of the substitutions were taken from [8].

Figure 1: (Colors online) Area under curves measuring the performance of the model in predicting antigenic relatedness for the four influenza A subtypes; H1N1 (blue with AUC value 0.79), H3N2 (red with AUC value 0.88), H5N1 (green with AUC value 0.90), and H1N1 (purple with AUC value 0.81).

Figure 2: (Colors online) Comparison plot between our model (blue bars) and the PREDAV-FLUA model (sky-blue bars) based on the accuracy metric for three influenza subtypes: H3N2, H5N1, and H1N1. The results of PREDAV-FLUA model were taken from [5].

Figure 3: (Colors online) Energy contribution from the five canonical epitopes of influenza A H3N2 viruses. This figure shows that the high efficiency neutralization epitopes (A, B, and D) relatively contribute a larger amount of energy than the low efficiency neutralization epitopes (C and E). The energy contribution for each epitope is sum of all the Hamiltonians corresponding to the amino acid positions allocated within the specific epitope and, to be comparable, we normalized the energy contribution for each epitope by dividing over its size.

Figure 4: (Colors online) Comparison of energy contribution from the five canonical epitopes (red); monoclonal and receptor binding sites (blue); and the consensus residues (green). The energy contribution for each category is sum of all the Hamiltonians corresponding to the amino acid positions allocated within the specific category.

Figure 5: (Colors online) Energy fluctuations over HA1 protein positions for H1N1 (green) H3N2 (red), H5N1 (blue), and H9N2 (black) influenza viruses.

Figure 6: (Colors online) The performance of the model on the validation dataset using two strategies: five fold cross validation test (red curve) and using the validation dataset as novel (unseen) dataset (blue curve). Both measured in terms AUC values.

Figure 7: ROC curve with AUC value = 0.80 from five-fold cross-validation test on dataset of all the four influenza A subtypes considered in this study.

References

[1] Qiu Jingxuan, Qiu Tianyi, Yang Yiyan, Wu Dingfeng, and Cao Zhiwei. Incorporating structure context of HA protein to improve antigenicity calculation for influenza virus A/H3N2. *Scientific Reports*, 6: 31156, jul 2016. ISSN 2045-2322. doi: 10.1038/srep31156.

[2] Ndifon Wilfred. New methods for analyzing serological data with applications to influenza surveillance. *Influenza and Other Respiratory Viruses*, 5(3):206–212, jan 2011. ISSN 1750-2640 1750-2659.

[3] Wilfred Ndifon, Ned S Wingreen, and Simon A Levin. Differential neutralization efficiency of hemagglutinin epitopes, antibody interference, and the design of influenza vaccines. *Proc Natl Acad Sci*, 106 (21):8701–8706, May 2009. doi: 10.1073/pnas.0903427106.

[4] Bui Huynh-Hoa, Peters Bjoern, Assarsson Erika, Mbawuike Innocent, and Sette Alessandro. Ab and T cell epitopes of influenza A virus, knowledge and opportunities. *Proceedings of the National Academy of Sciences of the United States of America*, 104(1):246–251, oct 2006. doi: 10.1073/pnas.0609330104.

[5] Peng Yousong, Wang Dayan, Wang Jianhong, Li Kenli, Tan Zhongyang, Shu Yuelong, and Jiang Taijiao. A universal computational model for predicting antigenic variants of influenza A virus based on conserved antigenic structures. *Scientific Report*, 7:42051, feb 2017. doi: 10.1038/srep4205110.1038/srep42051. URL `https://www.nature.com/articles/srep42051\#supplementary-information`.

[6] William D Lees, David S Moss, and Adrian J Shepherd. A computational analysis of the antigenic properties of haemagglutinin in influenza A H3N2. *Bioinformatics*, 26(11):1403–1408, 2010. doi: 10.1093/bioinformatics/btq160.

[7] Yoshiyuki Suzuki. Predictability of antigenic evolution for H3N2 human influenza A virus. *Genes & Genetic Systems*, 88(4):225–232, 2013. doi: 10.1266/ggs.88.225.

[8] Derek J Smith, Alan S Lapedes, Jan C de Jong, Theo M. Bestebroer, Guus F. Rimmelzwaan, Albert D. M. E. Osterhaus, and Ron A. M. Fouchier. Mapping the Antigenic and Genetic Evolution of Influenza Virus. *Science*, 305(5682):371–376, 2004. ISSN 0036-8075. doi: 10.1126/science.1097211.
